# Trends in Incidence and Premature Mortality (Years of Life Lost) From Acute Viral Hepatitis Among Children Under Five in Ethiopia, From 1990 to 2023: A Global Burden of Disease 2023 Study

**DOI:** 10.1155/ghe3/5287334

**Published:** 2026-08-12

**Authors:** Medhin Mehari, Jemal Bedewi, Zufan Tessema, Abadi Hailay Atsbaha, Alqeer Aliyo, Miesa Gelchu, Tibeso Gemechu, Yohannes Fekadu, Girma Ashenafi, Sebsibe Tadesse

**Affiliations:** ^1^ Department of Public Health, College of Medicine and Health Sciences, Adigrat University, Adigrat, Ethiopia, adu.edu.et; ^2^ Department of Public Health, College of Medicine and Health Sciences, Wolkite University, Wolkite, Ethiopia, wku.edu.et; ^3^ College of Medicine and Health Science, Wolkite University, Wolkite, Ethiopia, wku.edu.et; ^4^ Department of Medical Laboratory Science, Institute of Health, Bule Hora University, Bule Hora, Ethiopia, bhu.edu.et; ^5^ School of Public Health, Institute of Health, Bule Hora University, Bule Hora, Ethiopia, bhu.edu.et; ^6^ Department of Epidemiology, School of Public Health, Institute of Health, Bule Hora University, Bule Hora, Ethiopia, ufl.edu; ^7^ National Data Management and Analytics Center for Health, Ethiopian Public Health Institute, Addis Ababa, Ethiopia, ephi.gov.et

**Keywords:** acute viral hepatitis, children under five, Ethiopia, incidence, premature mortality, subnational disparities, trends

## Abstract

**Background:**

Globally, acute viral hepatitis (AVH) causes an estimated 1.5 million new infections annually among children under five, with sub‐Saharan Africa bearing a disproportionate burden. In Ethiopia, the Global Burden of Disease Study reported an age‐standardized incidence rate of 3616 per 100,000 population across all ages in 2019, with children under five being especially vulnerable to fecal–oral transmission. Despite available vaccines for Hepatitis B and preventive measures for Hepatitis A and E, comprehensive national data on long‐term trends and subnational disparities in this age group remained limited.

**Methods:**

We used data from the Global Burden of Disease (GBD) 2023 study to assess the burden of AVH among children under five in Ethiopia from 1990 to 2023. Estimates were disaggregated by region, sex, and hepatitis type. The burden of AVH was described in terms of incidence and years of life lost across all regions in Ethiopia from 1990 to 2023. Each metric was reported with numbers and rates per 100,000 population, along with 95% uncertainty intervals (UIs). Statistical significance was evaluated based on a 95% UI.

**Results:**

This study examined trends in AVH incidence and premature mortality among Ethiopian children under five from 1990 to 2023. The analysis revealed a statistically significant national decline in hepatitis B incidence, with an annualized rate of change of −0.68% (95% UI: −0.72, −0.63) per year. However, the overall acute hepatitis incidence showed only a modest decline of −0.07% (95% UI: −0.10, −0.05) per year, as declines in Hepatitis B were offset by stagnant rates of Hepatitis A and E. The most substantial regional decreases in Hepatitis B occurred in Addis Ababa and Southwest Ethiopia. Premature mortality, measured in years of life lost, also showed significant improvement, decreasing at an annual rate of −0.52% (95% UI: −0.79, −0.08) per year nationally. While these positive trends were observed, most variation between regions and across sexes did not reach statistical significance, indicating relatively consistent progress across different demographic groups and geographic areas.

**Conclusion:**

The observed decline in Hepatitis B incidence is likely attributable to the introduction of the Hepatitis B vaccination program, while the persistent burden of Hepatitis A and E probably reflects ongoing water, sanitation, and hygiene (WASH) challenges. To build on this progress and address persistent inequities, targeted vaccination campaigns in high‐burden regions must be combined with comprehensive water and sanitation improvements, alongside regionally adapted healthcare strategies.

**Evidence Before This Study:**

We searched PubMed, Embase, and the GBD repository for studies published from January 1, 1990, to December 31, 2023, using the terms “acute viral hepatitis,” “children,” “under‐five,” “incidence,” “mortality,” and “Ethiopia.” Previous studies were limited to localized, short‐term outbreaks or single‐center reports. No comprehensive national time‐trend analysis quantifying the long‐term burden and premature mortality of AVH in Ethiopian children under five was identified.

**Added Value of This Study:**

This is the first study to provide detailed, nationally representative estimates of the incidence and mortality due to AVH among children under five in Ethiopia over 33 years (1990–2023). We present subnational disparities, analyze trends, and examine the association with sociodemographic development.

**Implications of All the Available Evidence:**

The persistently high burden of AVH, driven largely by Hepatitis A and E, is a preventable cause of child mortality in Ethiopia. The evidence underscores the critical gap in preventive measures and calls for policy action integrating WASH improvements with vaccination strategy to achieve SDG targets for child survival.

## 1. Background

Acute viral hepatitis (AVH) refers to inflammation of the liver caused by a group of hepatotoxic viruses, primarily Hepatitis A, B, C, and E [1, 2], toxic substances, or autoimmune disorders [1, 3]. These infections can result in a wide spectrum of clinical manifestations ranging from asymptomatic to fulminant liver failure, particularly in young children [4, 5]. In Ethiopia, the burden of AVH remains a significant public health concern [6]. Based on estimates from the Global Burden of Disease (GBD) 2023 study, Ethiopia has experienced considerable incidence and premature mortality attributed to AVH among children under five [4, 7]. Years of life lost (YLLs), a measure of premature mortality, continue to reflect the severity of hepatitis‐related deaths in this vulnerable age group [8].

In response to the burden of hepatitis, various regions in Ethiopia have developed national health strategies and intervention programs, including routine childhood vaccination for Hepatitis B, promotion of sanitation and hygiene to prevent Hepatitis A and E, and blood screening practices for Hepatitis C [9]. Despite these efforts, AVH remains underreported and under‐prioritized in the health agendas of many countries in the region. A critical gap exists in understanding subnational trends and variations in incidence and mortality, particularly among under‐five children. These gaps limit the capacity of policymakers and program implementers to allocate resources effectively and tailor interventions to areas of greatest need.

A critical gap exists in understanding subnational trends and variations in incidence and mortality, particularly among children under five. While previous studies have reported regional estimates for Ethiopia [10, 11], none have provided a comprehensive 33‐year trend analysis disaggregated by region, sex, and hepatitis type specifically for children under five. Prior estimates have been limited to all‐age populations or short‐term outbreak reports, leaving uncertainty about whether national averages mask important subnational disparities that could inform targeted interventions.

Therefore, this study aims to determine the trends of incidence and premature mortality from AVH among children under five by sex, age, and location in Ethiopia from 1990 to 2023 using data, methods, and tools of the GBD 2023 study. The findings from this study will offer valuable evidence for national and subnational health authorities, development partners, and other key stakeholders to strengthen surveillance, refine prevention strategies, and accelerate progress toward eliminating viral hepatitis as a public health threat in the region [12].

## 2. Methods

### 2.1. Study Setting

This study was conducted in Ethiopia’s different regions, which include Amhara, Tigray, Afar, Oromia, Gambella, Harari, Somali, Sidama, Benishangul‐Gumuz, Southwest Ethiopia, and the Southern Nations, Nationalities, and Peoples’ (SNNP). Additionally, two chartered city administrations (Addis Ababa and Dire Dawa) were included. For consistency across the 1990–2023 time series, we used the regional classifications exactly as defined in the GBD 2023 study. In 2019, Ethiopia had an estimated age‐standardized incidence rate of 3616 per 100,000 person‐years for AVH across all ages [11, 13]. The current study focuses specifically on the burden among children under five.

### 2.2. Data Sources

The GBD 2023 study data were used to estimate the incidence and premature mortality in Ethiopia from 1990 to 2023. These data include national health surveys, verbal autopsies, and hospital records data (https://ghdx.healthdata.org/gbd-2023/sources).

### 2.3. Data Analysis

YLLs, a measure of premature mortality, were estimated using the cause of death ensemble model (CODEm). YLLs were determined by multiplying the number of deaths from AVH by the life expectancy at the age of death [13, 14]. Disease modeling with the Bayesian meta‐regression tool (DisMod‐MR 2.1) was used to estimate the incidence of acute hepatitis.

To quantify the magnitude and direction of changes over the 33‐year study period, we calculated the annualized rate of change (ARC) using the standard GBD methodology (GBD 2023 Disease and Injury and Risk Factor Collaborators, 2025). The ARC represents the average annual percent change in the rate over the study period, estimated by fitting a linear regression model to the natural logarithm of the rates. Specifically, the model is defined as ln(rate) = *α* + *β* × year, where *β* represents the regression coefficient. The ARC was then calculated as (exp(*β*) – 1) × 100, representing the average annual percent change [15]. This regression‐based approach captures the overall trend across the entire period, rather than relying solely on endpoint‐to‐endpoint comparisons, and accounts for year‐to‐year variability.

Trends were considered statistically significant if the 95% uncertainty interval (UI) for the ARC did not include zero [16, 17]. This indicates that the observed change is unlikely to be due to random variation. We analyzed the burden of acute hepatitis across three nested age groups relevant to childhood hepatitis epidemiology: neonates (0–27 days), infants (< 1 year), and children under five (0–4 years). The GBD study provides estimates for each of these age strata separately, enabling age‐specific analyses that are important for targeting interventions.

### 2.4. Ethics

This study used publicly available, secondary data from the GBD 2023 study. Moreover, the estimates were aggregated at the population level so that individual‐level data were adequately anonymized to ensure confidentiality of information. Therefore, ethical consent for human participation was not required for this study. This study was conducted as part of the GBD Collaborators Network and adhered to the GBD protocol (https://www.healthdata.org/research-analysis/about-gbd/protocol).

## 3. Results

### 3.1. Incidence of Acute Viral Hepatitis Among Under‐Five Children

**FIGURE 1:**
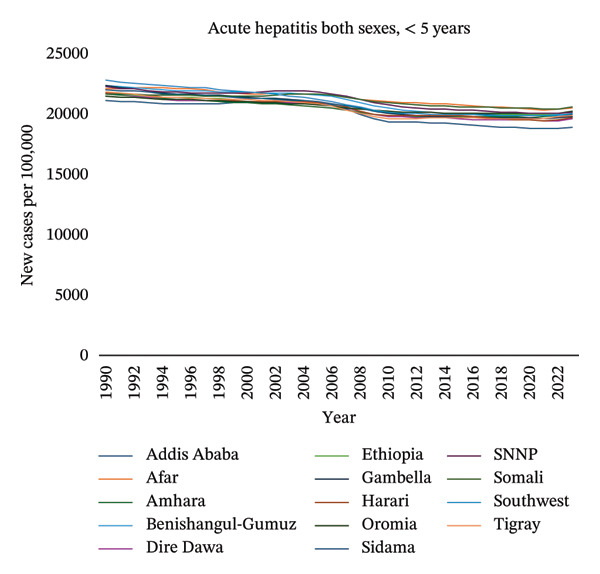
Incidence rate of acute hepatitis among children under five in Ethiopia, 1990 to 2023.

There were an estimated 3,301,740 new cases (95% UI: 3,022,150, 3,497,257) of AVH among children under five in Ethiopia in 2023. The incidence was fairly balanced between sexes, with 1,687,624 new male cases (95% UI: 1,546,421, 1,788,690) and 1,614,116 female cases (95% UI: 1,475,304, 1,713,342). The equivalent incidence rate was 20,130 new cases per 100,000 population (95% UI: 18,425, 21,322), with 20,101 new cases per 100,000 males (95% UI: 18,420, 21,305) and 20,160 new cases per 100,000 females (95% UI: 18,426, 21,399). The 95% UIs for regional estimates overlapped. There was a −0.07% decline (95% UI: −0.10, −0.05) per year in the national incidence rate from 1990 to 2023, with slight subnational differences (Table 1, Figure 1).

**TABLE 1:** Incidence rate of acute viral hepatitis among children under five by sex and location in Ethiopia, 1990 to 2023.

Location	Sex	Acute hepatitis	Annualized rate of change	Hepatitis A	Annualized rate of change	Hepatitis B	Annualized rate of change	Hepatitis C	Annualized rate of change	Hepatitis E	Annualized rate of change
		2023	1990–2023	2023	1990–2023	2023	1990–2023	2023	1990–2023	2023	1990–2023
Addis Ababa	Male	18,894 (17,139, 20,436)	−0.11 (−0.18, −0.06)	17,536 (15,739, 18,997)	−0.05 (−0.12, 0.02)	201 (151, 260)	−0.88 (−0.91, −0.85)	48 (35, 62)	−0.15 (−0.30, 0.05)	1109 (825, 1336)	0.00 (−0.11, 0.14)
	Female	18,853 (17,097, 20,359)	−0.10 (−0.16, −0.04)	17,496 (15,755, 18,986)	−0.05 (−0.12, 0.01)	159 (117, 206)	−0.87 (−0.90, −0.83)	50 (36, 64)	−0.28 (−0.46, −0.11)	1149 (846, 1401)	0.00 (−0.12, 0.12)
	Both	18,874 (17,209, 20,298)	−0.11 (−0.15, −0.06)	17,516 (15,782, 18,883)	−0.05 (−0.10, 0.00)	180 (139, 232)	−0.88 (−0.90, −0.85)	49 (37, 62)	−0.22 (−0.34, −0.08)	1128 (841, 1357)	0.00 (−0.08, 0.10)

Afar	Male	20,486 (18,587, 21,877)	−0.06 (−0.12, 0.00)	18,401 (16,573, 19,744)	−0.02 (−0.08, 0.05)	929 (697, 1226)	−0.51 (−0.67, −0.26)	62 (43, 81)	−0.11 (−0.27, 0.09)	1093 (779, 1345)	0.00 (−0.13, 0.15)
	Female	20,631 (18,828, 22,043)	−0.08 (−0.14, −0.03)	18,570 (16,774, 19,833)	−0.04 (−0.10, 0.01)	805 (598, 1087)	−0.57 (−0.69, −0.40)	84 (69, 94)	−0.03 (−0.20, 0.15)	1173 (859, 1423)	0.01 (−0.12, 0.16)
	Both	20,554 (18,772, 21,921)	−0.07 (−0.11, −0.03)	18,481 (16,790, 19,713)	−0.03 (−0.07, 0.02)	870 (677, 1108)	−0.54 (−0.63, −0.43)	72 (59, 85)	−0.06 (−0.18, 0.05)	1131 (848, 1368)	0.01 (−0.09, 0.09)

Amhara	Male	19,957 (17,976, 21,234)	−0.08 (−0.14, −0.03)	18,476 (16,538, 19,733)	−0.04 (−0.11, 0.03)	339 (259, 457)	−0.76 (−0.82, −0.67)	52 (38, 69)	−0.17 (−0.33, 0.02)	1089 (775, 1327)	−0.01 (−0.14, 0.12)
	Female	19,956 (18,260, 21,351)	−0.08 (−0.14, −0.01)	18,455 (16,748, 19,712)	−0.04 (−0.09, 0.03)	266 (187, 366)	−0.78 (−0.86, −0.68)	71 (56, 87)	−0.14 (−0.28, 0.04)	1163 (855, 1399)	0.00 (−0.12, 0.14)
	Both	19,956 (18,095, 21,161)	−0.08 (−0.12, −0.04)	18,466 (16,697, 19,573)	−0.04 (−0.09, 0.00)	303 (227, 398)	−0.77 (−0.83, −0.69)	62 (49, 74)	−0.15 (−0.27, 0.00)	1125 (818, 1353)	0.00 (−0.10, 0.09)

Benishangul‐Gumuz	Male	19,971 (18,075, 21,233)	−0.10 (−0.16, −0.04)	18,558 (16,766, 19,819)	−0.04 (−0.09, 0.03)	266 (195, 361)	−0.86 (−0.90, −0.81)	55 (41, 71)	−0.20 (−0.37, −0.04)	1092 (819, 1360)	−0.01 (−0.12, 0.12)
	Female	19,996 (18,134, 21,350)	−0.11 (−0.17, −0.06)	18,531 (16,732, 19,809)	−0.04 (−0.11, 0.02)	237 (165, 320)	−0.88 (−0.91, −0.83)	66 (49, 82)	−0.20 (−0.38, 0.01)	1162 (911, 1426)	−0.01 (−0.13, 0.16)
	Both	19,983 (18,362, 21,173)	−0.11 (−0.15, −0.07)	18,545 (17,000, 19,699)	−0.04 (−0.08, 0.00)	252 (186, 322)	−0.87 (−0.90, −0.83)	61 (46, 75)	−0.20 (−0.34, −0.07)	1126 (862, 1367)	−0.01 (−0.09, 0.10)

Dire Dawa	Male	19,562 (17,662, 20,892)	−0.10 (−0.16, −0.04)	18,229 (16,430, 19,471)	−0.05 (−0.12, 0.02)	184 (141, 239)	−0.87 (−0.90, −0.82)	49 (35, 61)	−0.22 (−0.36, −0.04)	1100 (809, 1348)	0.00 (−0.11, 0.19)
	Female	19,652 (17,711, 20,991)	−0.09 (−0.15, −0.04)	18,282 (16,468, 19,548)	−0.05 (−0.11, 0.01)	156 (109, 219)	−0.87 (−0.90, −0.79)	54 (39, 66)	−0.30 (−0.44, −0.13)	1161 (861, 1436)	−0.01 (−0.13, 0.12)
	Both	19,606 (17,754, 20,856)	−0.10 (−0.14, −0.06)	18,254 (16,582, 19,339)	−0.05 (−0.10, −0.01)	170 (134, 216)	−0.87 (−0.89, −0.84)	51 (38, 63)	−0.26 (−0.37, −0.15)	1130 (840, 1369)	0.00 (−0.08, 0.10)

Ethiopia	Male	20,101 (18,420, 21,305)	−0.08 (−0.11, −0.05)	18,486 (16,831, 19,673)	−0.03 (−0.06, −0.01)	463 (335, 583)	−0.69 (−0.75, −0.62)	55 (42, 68)	−0.14 (−0.26, −0.04)	1097 (827, 1307)	0.00 (−0.06, 0.07)
	Female	20,160 (18,426, 21,399)	−0.07 (−0.11, −0.03)	18,491 (16,760, 19,639)	−0.04 (−0.08, 0.01)	439 (338, 549)	−0.66 (−0.73, −0.60)	66 (56, 76)	−0.15 (−0.23, −0.07)	1164 (874, 1406)	0.00 (−0.07, 0.07)
	Both	20,130 (18,425, 21,322)	−0.07 (−0.10, −0.05)	18,489 (16,793, 19,572)	−0.03 (−0.06, −0.01)	451 (355, 550)	−0.68 (−0.72, −0.63)	60 (48, 71)	−0.15 (−0.21, −0.07)	1129 (850, 1346)	0.00 (−0.05, 0.05)

Gambella	Male	19,840 (18,199, 21,168)	−0.12 (−0.17, −0.06)	18,298 (16,753, 19,607)	−0.06 (−0.13, 0.01)	389 (278, 510)	−0.79 (−0.85, −0.71)	55 (37, 74)	−0.19 (−0.36, 0.00)	1097 (802, 1330)	0.01 (−0.14, 0.15)
	Female	19,840 (17,913, 21,199)	−0.11 (−0.17, −0.05)	18,347 (16,506, 19,670)	−0.06 (−0.12, 0.01)	275 (200, 362)	−0.82 (−0.86, −0.75)	53 (40, 68)	−0.32 (−0.45, −0.16)	1165 (829, 1430)	0.00 (−0.14, 0.13)
	Both	19,840 (18,203, 21,152)	−0.11 (−0.15, −0.07)	18,322 (16,772, 19,594)	−0.06 (−0.11, −0.01)	333 (249, 419)	−0.80 (−0.84, −0.75)	54 (40, 69)	−0.26 (−0.36, −0.13)	1130 (839, 1362)	0.00 (−0.09, 0.10)

Harari	Male	19,707 (17,868, 21,123)	−0.09 (−0.15, −0.04)	18,046 (16,199, 19,378)	−0.05 (−0.11, 0.01)	492 (376, 626)	−0.69 (−0.78, −0.59)	59 (43, 80)	−0.13 (−0.30, 0.05)	1109 (794, 1359)	0.01 (−0.11, 0.16)
	Female	19,687 (17,862, 20,994)	−0.09 (−0.15, −0.04)	18,075 (16,321, 19,431)	−0.06 (−0.12, 0.00)	380 (291, 491)	−0.71 (−0.78, −0.53)	59 (41, 75)	−0.21 (−0.42, −0.02)	1173 (885, 1425)	0.01 (−0.12, 0.20)
	Both	19,697 (17,917, 21,021)	−0.09 (−0.14, −0.06)	18,060 (16,383, 19,304)	−0.05 (−0.10, −0.01)	438 (347, 553)	−0.70 (−0.75, −0.65)	59 (43, 76)	−0.17 (−0.33, −0.02)	1140 (867, 1375)	0.01 (−0.08, 0.12)

Oromia	Male	20,184 (18,449, 21,501)	−0.06 (−0.12, −0.01)	18,531 (16,802, 19,727)	−0.03 (−0.10, 0.03)	500 (324, 683)	−0.60 (−0.69, −0.48)	54 (40, 69)	−0.14 (−0.31, 0.04)	1099 (815, 1331)	0.01 (−0.11, 0.22)
	Female	20,240 (18,516, 21,603)	−0.05 (−0.12, 0.01)	18,495 (16,772, 19,761)	−0.03 (−0.10, 0.04)	532 (385, 694)	−0.50 (−0.64, −0.31)	52 (38, 69)	−0.23 (−0.41, −0.06)	1161 (843, 1403)	−0.01 (−0.14, 0.14)
	Both	20,211 (18,561, 21,442)	−0.06 (−0.10, −0.02)	18,513 (16,863, 19,689)	−0.03 (−0.07, 0.01)	516 (387, 658)	−0.55 (−0.63, −0.47)	53 (40, 66)	−0.19 (−0.28, −0.05)	1129 (836, 1359)	0.00 (−0.09, 0.12)

Sidama	Male	19,914 (18,013, 21,307)	−0.10 (−0.16, −0.05)	18,407 (16,637, 19,761)	−0.03 (−0.10, 0.02)	341 (255, 445)	−0.82 (−0.86, −0.76)	66 (48, 85)	−0.06 (−0.22, 0.14)	1100 (817, 1331)	0.01 (−0.12, 0.15)
	Female	20,032 (18,409, 21,359)	−0.09 (−0.14, −0.03)	18,472 (16,881, 19,653)	−0.04 (−0.10, 0.03)	305 (216, 396)	−0.81 (−0.87, −0.75)	80 (62, 94)	−0.02 (−0.19, 0.25)	1174 (890, 1434)	0.01 (−0.12, 0.15)
	Both	19,972 (18,358, 21,252)	−0.09 (−0.13, −0.06)	18,439 (16,847, 19,621)	−0.04 (−0.08, 0.01)	324 (250, 412)	−0.81 (−0.86, −0.76)	73 (57, 88)	−0.04 (−0.16, 0.13)	1136 (850, 1365)	0.01 (−0.07, 0.12)

Somali	Male	20,567 (18,744, 21,942)	−0.05 (−0.10, 0.01)	18,530 (16,814, 19,815)	−0.01 (−0.06, 0.05)	886 (638, 1175)	−0.46 (−0.61, −0.31)	58 (40, 76)	−0.09 (−0.25, 0.15)	1092 (782, 1332)	0.00 (−0.12, 0.13)
	Female	20,640 (18,796, 22,157)	−0.06 (−0.11, 0.00)	18,622 (16,797, 19,930)	−0.02 (−0.08, 0.03)	776 (540, 1121)	−0.52 (−0.71, −0.28)	78 (62, 90)	−0.03 (−0.20, 0.22)	1163 (858, 1416)	−0.01 (−0.14, 0.11)
	Both	20,602 (18,944, 21,992)	−0.05 (−0.09, −0.01)	18,574 (16,904, 19,755)	−0.02 (−0.06, 0.02)	833 (614, 1114)	−0.49 (−0.63, −0.35)	68 (55, 80)	−0.05 (−0.18, 0.11)	1126 (836, 1350)	0.00 (−0.11, 0.08)

Southwest	Male	19,984 (18,058, 21,416)	−0.13 (−0.18, −0.08)	18,507 (16,705, 19,889)	−0.06 (−0.12, 0.00)	315 (251, 404)	−0.85 (−0.89, −0.79)	63 (46, 80)	−0.12 (−0.29, 0.09)	1099 (817, 1327)	0.01 (−0.11, 0.15)
	Female	20,092 (18,382, 21,452)	−0.12 (−0.17, −0.06)	18,560 (16,937, 19,762)	−0.06 (−0.12, 0.01)	281 (210, 374)	−0.84 (−0.88, −0.80)	78 (59, 94)	−0.01 (−0.20, 0.21)	1173 (882, 1431)	0.01 (−0.12, 0.14)
	Both	20,037 (18,414, 21,298)	−0.12 (−0.16, −0.08)	18,533 (16,910, 19,742)	−0.06 (−0.10, −0.01)	298 (237, 380)	−0.84 (−0.88, −0.80)	71 (56, 83)	−0.06 (−0.20, 0.10)	1135 (851, 1359)	0.01 (−0.08, 0.12)

SNNP	Male	20,055 (18,150, 21,457)	−0.10 (−0.16, −0.06)	18,517 (16,713, 19,898)	−0.04 (−0.10, 0.01)	383 (287, 507)	−0.80 (−0.87, −0.71)	58 (44, 76)	−0.14 (−0.30, 0.09)	1098 (815, 1329)	0.01 (−0.12, 0.15)
	Female	20,203 (18,468, 21,510)	−0.09 (−0.14, −0.04)	18,592 (16,964, 19,796)	−0.04 (−0.10, 0.03)	352 (263, 506)	−0.77 (−0.83, −0.68)	87 (76, 95)	0.02 (−0.15, 0.24)	1172 (890, 1431)	0.01 (−0.12, 0.14)
	Both	20,128 (18,514, 21,402)	−0.10 (−0.14, −0.07)	18,553 (16,926, 19,767)	−0.04 (−0.09, 0.01)	368 (294, 467)	−0.79 (−0.84, −0.72)	73 (63, 83)	−0.05 (−0.17, 0.09)	1134 (848, 1361)	0.01 (−0.07, 0.12)

Tigray	Male	19,870 (18,018, 21,166)	−0.09 (−0.15, −0.02)	18,375 (16,624, 19,668)	−0.03 (−0.10, 0.05)	345 (257, 445)	−0.79 (−0.83, −0.72)	49 (35, 64)	−0.23 (−0.39, −0.05)	1101 (825, 1364)	0.01 (−0.13, 0.14)
	Female	19,878 (17,962, 21,282)	−0.09 (−0.14, −0.04)	18,382 (16,598, 19,692)	−0.04 (−0.09, 0.01)	277 (201, 393)	−0.82 (−0.86, −0.77)	59 (45, 73)	−0.27 (−0.44, −0.11)	1160 (881, 1411)	−0.01 (−0.13, 0.15)
	Both	19,874 (18,062, 21,082)	−0.09 (−0.13, −0.05)	18,378 (16,685, 19,555)	−0.04 (−0.08, 0.01)	312 (230, 409)	−0.80 (−0.83, −0.77)	54 (40, 67)	−0.25 (−0.38, −0.14)	1130 (868, 1353)	0.00 (−0.10, 0.10)

Hepatitis A accounted for an estimated 18,489 new cases per 100,000 children under five (95% UI: 16,793, 19,572) nationally in 2023. The point estimates were similar, with overlapping UIs between sexes and across subnational regions (Table 1). From 1990 to 2023, there were slight and statistically significant declines in the incidence rate nationally, with the largest reductions occurring in Gambella and Southwest (Table 1).

Hepatitis B incidence was estimated at 451 new cases per 100,000 children under five (95% UI: 355, 550) nationally in 2023. Afar (870 new cases per 100,000 children under five [95% UI: 677, 1108]) and Somali (833 new cases per 100,000 children under five [95% UI: 614, 1114]) regions had higher, while Addis Ababa (180 [95% UI: 139, 232]), Dire Dawa (170 new cases per 100,000 children under five [95% UI: 134, 216]), and Benishangul‐Gumuz (252 new cases per 100,000 children under five [95% UI: 186, 322]) had the lowest. A significant decline in the incidence rate nationally −0.68% decline (95% UI: −0.72, −0.63) per year was observed from 1990 to 2023, with substantial regional variation (Table 1).

Hepatitis C incidence was 60 new cases per 100,000 children under five (95% UI: 48, 71) nationally in 2023, with similar rates across subnational regions (Table 1). A slight but statistically significant decline was observed nationally from 1990 to 2023, with an ARC of −0.15% (95% UI: −0.21, −0.07) per year (Table 1).

Hepatitis E accounted for 1129 new cases per 100,000 children under five (95% UI: 850, 1346) nationally in 2023. The 95% UIs for sex‐specific estimates overlapped substantially. No trend changes were observed from 1990 to 2023 (Table 1).

### 3.2. Incidence of Acute Viral Hepatitis Among Infants and Neonates

There were an estimated 35,794 new cases of AVH (95% UI: 29,107, 40,649) among 100,000 infants in Ethiopia in 2023 and 68,023 new cases (95% UI: 52,468, 80,236) among 100,000 neonates.

**FIGURE 2:**
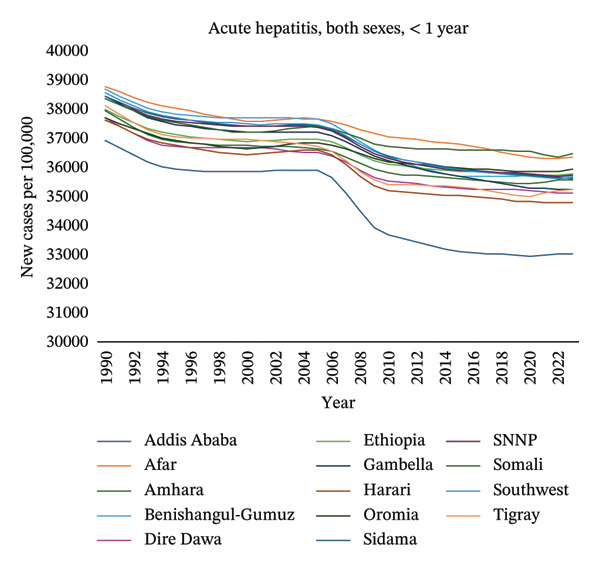
Incidence rate of acute hepatitis among infants in Ethiopia, 1990 to 2023.

**FIGURE 3:**
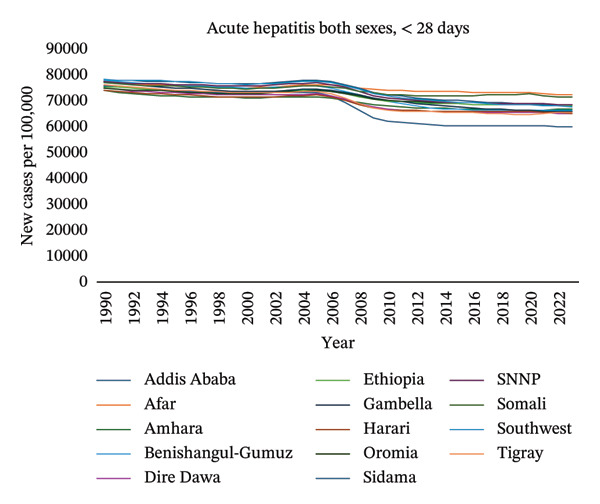
Incidence rate of acute hepatitis among neonates in Ethiopia, 1990 to 2023.

From 1990 to 2023, there was a slight decline in the incidence rate nationally for infants, with an ARC of ‐0.06% (95% UI: −0.11, −0.01) per year, and for neonates, with an ARC of −0.09% (95% UI: −0.17, −0.03) per year. For Hepatitis A and E, rates remained largely unchanged across both age groups. Hepatitis B showed significant declines in both infants, with an ARC of −0.65% (95% UI: −0.70, −0.61) per year, and neonates, with an ARC of −0.56% (95% UI: −0.62, −0.51) per year, with Afar and Somali consistently having higher rates than other regions. Detailed estimates for all hepatitis types and regions are presented in Table 2 (Figures 2 and 3).

**TABLE 2:** Incidence rate of AVH among infants and neonates by location in Ethiopia, 1990 to 2023.

Location	Age	Acute hepatitis	Annualized rate of change	Hepatitis A	Annualized rate of change	Hepatitis B	Annualized rate of change	Hepatitis C	Annualized rate of change	Hepatitis E	Annualized rate of change
		2023	1990–2023	2023	1990–2023	2023	1990–2023	2023	1990–2023	2023	1990–2023
Addis Ababa	< 1 year	33,038 (26,217, 39,579)	−0.11 (−0.22, −0.01)	31,673 (24,782, 38,110)	−0.06 (−0.19, 0.05)	225 (183, 295)	−0.88 (−0.90, −0.85)	43 (31, 57)	−0.24 (−0.37, −0.09)	1096 (693, 1563)	0.00 (−0.11, 0.12)
	< 28 days	59,899 (44,096, 75,865)	−0.20 (−0.32, −0.08)	57,006 (41,286, 73,089)	−0.08 (−0.23, 0.09)	998 (816, 1191)	−0.90 (−0.91, −0.88)	40 (26, 55)	−0.25 (−0.39, −0.08)	1855 (1074, 2923)	−0.02 (−0.13, 0.11)

Afar	< 1 year	36,378 (29,525, 41,712)	−0.06 (−0.16, 0.03)	33,926 (27,090, 39,202)	−0.04 (−0.13, 0.07)	1262 (1027, 1645)	−0.50 (−0.58, −0.40)	66 (53, 79)	−0.06 (−0.19, 0.06)	1123 (710, 1611)	0.00 (−0.13, 0.14)
	< 28 days	72,170 (55,957, 86,150)	−0.07 (−0.18, 0.04)	62,042 (45,923, 75,558)	−0.04 (−0.16, 0.10)	7874 (6401, 9524)	−0.34 (−0.44, −0.23)	63 (49, 77)	−0.06 (−0.18, 0.08)	2191 (1281, 3424)	0.03 (−0.12, 0.18)

Amhara	< 1 year	35,597 (28,178, 40,392)	−0.06 (−0.17, 0.03)	34,030 (26,808, 38,961)	−0.03 (−0.16, 0.07)	412 (322, 554)	−0.73 (−0.79, −0.67)	57 (45, 69)	−0.15 (−0.28, −0.01)	1098 (700, 1507)	−0.02 (−0.14, 0.12)
	< 28 days	66,639 (50,053, 79,669)	−0.09 (−0.22, 0.04)	62,242 (46,179, 75,048)	−0.03 (−0.18, 0.10)	2432 (1929, 2863)	−0.62 (−0.69, −0.53)	54 (42, 66)	−0.15 (−0.28, 0.01)	1911 (1174, 2959)	−0.04 (−0.18, 0.12)

Benishangul‐Gumuz	< 1 year	35,696 (28,968, 40,447)	−0.08 (−0.17, 0.01)	34,238 (27,644, 39,109)	−0.03 (−0.12, 0.07)	312 (244, 402)	−0.86 (−0.89, −0.83)	54 (39, 68)	−0.22 (−0.37, −0.08)	1093 (693, 1540)	−0.01 (−0.12, 0.14)
	< 28 days	66,068 (50,765, 78,842)	−0.14 (−0.25, −0.04)	62,684 (47,043, 75,682)	−0.03 (−0.16, 0.10)	1523 (1256, 1850)	−0.83 (−0.85, −0.81)	50 (34, 65)	−0.23 (−0.37, −0.07)	1812 (1051, 2763)	−0.02 (−0.15, 0.14)

Dire Dawa	< 1 year	35,128 (28,017, 40,603)	−0.07 (−0.17, 0.01)	33,749 (26,875, 39,105)	−0.03 (−0.15, 0.06)	226 (188, 288)	−0.86 (−0.88, −0.84)	46 (32, 56)	−0.28 (−0.40, −0.16)	1107 (695, 1572)	−0.01 (−0.12, 0.11)
	< 28 days	64,845 (49,369, 77,901)	−0.13 (−0.26, −0.02)	61,587 (46,635, 74,200)	−0.04 (−0.19, 0.10)	1289 (1049, 1525)	−0.84 (−0.86, −0.81)	43 (28, 55)	−0.28 (−0.41, −0.16)	1926 (1126, 2941)	−0.04 (−0.16, 0.09)

Ethiopia	< 1 year	35,794 (29,107, 40,649)	−0.06 (−0.11, −0.01)	33,992 (27,464, 38,788)	−0.03 (−0.09, 0.02)	640 (518, 806)	−0.65 (−0.70, −0.61)	55 (42, 67)	−0.15 (−0.22, −0.07)	1107 (720, 1536)	−0.01 (−0.08, 0.05)
	< 28 days	68,023 (52,468, 80,236)	−0.09 (−0.17, −0.03)	62,114 (46,811, 73,773)	−0.03 (−0.11, 0.04)	3862 (3176, 4495)	−0.56 (−0.62, −0.51)	51 (38, 65)	−0.15 (−0.22, −0.06)	1996 (1138, 3050)	−0.02 (−0.10, 0.04)

Gambella	< 1 year	35,261 (28,816, 40,633)	−0.08 (−0.19, 0.01)	33,664 (27,180, 38,972)	−0.04 (−0.16, 0.06)	446 (354, 586)	−0.79 (−0.83, −0.75)	48 (35, 63)	−0.27 (−0.37, −0.15)	1103 (681, 1549)	−0.01 (−0.14, 0.16)
	< 28 days	65,759 (51,060, 79,521)	−0.14 (−0.26, −0.03)	61,346 (46,764, 74,854)	−0.05 (−0.19, 0.09)	2427 (1973, 2910)	−0.75 (−0.79, −0.71)	45 (30, 60)	−0.28 (−0.38, −0.16)	1942 (1123, 2990)	−0.03 (−0.18, 0.16)

Harari	< 1 year	34,792 (27,613, 40,248)	−0.08 (−0.20, 0.01)	33,065 (25,955, 38,617)	−0.05 (−0.17, 0.04)	552 (449, 716)	−0.70 (−0.74, −0.65)	51 (35, 67)	−0.20 (−0.33, −0.06)	1123 (707, 1600)	0.00 (−0.12, 0.13)
	< 28 days	65,033 (48,901, 79,412)	−0.12 (−0.26, −0.01)	60,044 (43,677, 74,341)	−0.05 (−0.21, 0.08)	2837 (2327, 3411)	−0.68 (−0.71, −0.64)	48 (31, 64)	−0.21 (−0.33, −0.04)	2104 (1272, 3334)	−0.02 (−0.16, 0.13)

Oromia	< 1 year	35,940 (29,369, 41,154)	−0.05 (−0.14, 0.03)	34,075 (27,409, 39,080)	−0.03 (−0.12, 0.06)	712 (565, 889)	−0.54 (−0.60, −0.48)	47 (32, 61)	−0.20 (−0.30, −0.07)	1107 (686, 1538)	−0.01 (−0.13, 0.13)
	< 28 days	68,384 (52,678, 80,963)	−0.08 (−0.18, 0.02)	62,298 (46,704, 75,003)	−0.03 (−0.16, 0.09)	4059 (3380, 4734)	−0.51 (−0.59, −0.43)	43 (28, 60)	−0.20 (−0.34, −0.04)	1983 (1125, 3086)	−0.01 (−0.14, 0.13)

Sidama	< 1 year	35,617 (28,806, 40,701)	−0.07 (−0.17, 0.01)	33,894 (26,948, 38,897)	−0.03 (−0.13, 0.08)	532 (434, 689)	−0.79 (−0.82, −0.74)	67 (51, 83)	−0.03 (−0.17, 0.14)	1124 (712, 1590)	0.00 (−0.12, 0.12)
	< 28 days	68,160 (52,011, 80,682)	−0.13 (−0.24, 0.00)	61,986 (45,130, 74,506)	−0.03 (−0.16, 0.13)	3872 (3016, 4718)	−0.72 (−0.76, −0.66)	63 (48, 79)	−0.02 (−0.19, 0.19)	2238 (1256, 3411)	−0.01 (−0.15, 0.14)

Somali	< 1 year	36,467 (29,566, 41,591)	−0.05 (−0.14, 0.03)	34,115 (27,520, 39,225)	−0.03 (−0.12, 0.06)	1179 (936, 1475)	−0.46 (−0.58, −0.33)	62 (48, 75)	−0.05 (−0.18, 0.10)	1110 (709, 1546)	0.00 (−0.14, 0.13)
	< 28 days	71,269 (55,630, 84,005)	−0.07 (−0.17, 0.04)	62,390 (47,018, 75,008)	−0.03 (−0.15, 0.10)	6777 (5507, 8039)	−0.34 (−0.42, −0.24)	59 (45, 72)	−0.05 (−0.18, 0.11)	2043 (1218, 3170)	0.00 (−0.15, 0.13)

Southwest	< 1 year	35,658 (28,792, 40,762)	−0.08 (−0.17, 0.01)	33,994 (27,016, 39,026)	−0.03 (−0.14, 0.07)	481 (397, 589)	−0.82 (−0.85, −0.78)	65 (50, 78)	−0.05 (−0.21, 0.13)	1118 (708, 1581)	−0.01 (−0.13, 0.12)
	< 28 days	67,605 (51,344, 80,044)	−0.14 (−0.25, −0.02)	61,976 (45,123, 74,495)	−0.03 (−0.16, 0.13)	3434 (2658, 4049)	−0.74 (−0.78, −0.69)	62 (46, 76)	−0.04 (−0.21, 0.16)	2134 (1198, 3250)	−0.04 (−0.18, 0.11)

SNNP	< 1 year	35,746 (28,909, 40,798)	−0.07 (−0.16, 0.02)	33,975 (27,003, 39,004)	−0.03 (−0.13, 0.08)	589 (458, 754)	−0.75 (−0.82, −0.68)	68 (57, 77)	−0.04 (−0.16, 0.13)	1114 (704, 1575)	0.00 (−0.12, 0.12)
	< 28 days	68,541 (52,301, 81,288)	−0.11 (−0.22, 0.01)	61,977 (45,125, 74,498)	−0.03 (−0.16, 0.13)	4420 (3353, 5581)	−0.62 (−0.71, −0.52)	65 (54, 75)	−0.03 (−0.17, 0.15)	2079 (1168, 3166)	0.00 (−0.08, 0.12)

Tigray	< 1 year	35,259 (27,819, 40,234)	−0.08 (−0.18, 0.02)	33,674 (26,509, 38,665)	−0.03 (−0.15, 0.08)	393 (311, 513)	−0.80 (−0.82, −0.76)	49 (35, 62)	−0.27 (−0.38, −0.14)	1100 (712, 1529)	−0.01 (−0.11, 0.12)
	< 28 days	65,255 (48,483, 78,560)	−0.14 (−0.27, −0.02)	61,527 (44,877, 74,811)	−0.04 (−0.19, 0.10)	1823 (1481, 2214)	−0.79 (−0.82, −0.75)	46 (31, 60)	−0.27 (−0.40, −0.14)	1859 (1050, 2865)	−0.02 (−0.14, 0.11)

### 3.3. Premature Mortality From AVH Among Under‐Five Children

**FIGURE 4:**
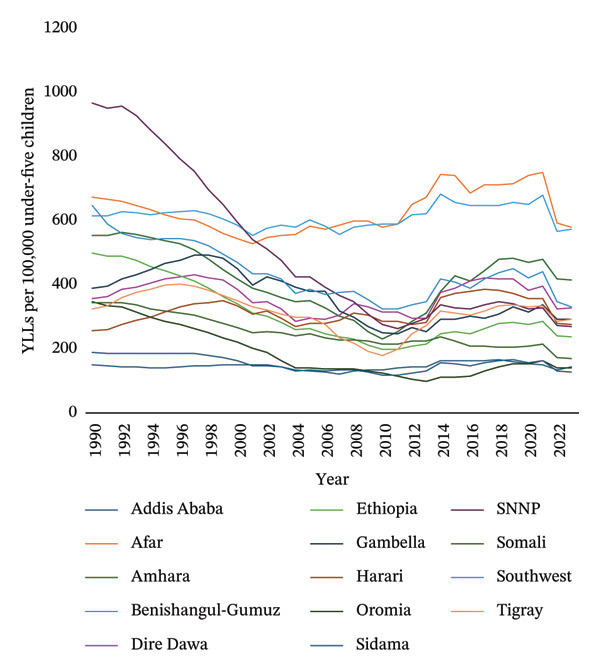
YLL rate of acute hepatitis among children under five in Ethiopia, 1990 to 2023.

There were an estimated 239 YLLs per 100,000 children under five (95% UI: 129, 425) from AVH in Ethiopia in 2023, with 286 YLLs per 100,000 male children (95% UI: 117, 564) and 190 per 100,000 female children (95% UI: 66, 377). In the same year, the 95% UIs for YLL estimates overlapped substantially. From 1990 to 2023, there were slight trend changes in the YLL rate in Oromia [a 0.59% decline (95% UI: −0.83, −0.09)] and SNNP [a 0.72% decline (95% UI: −0.88, −0.33)] (Table 3, Figure 4).

**TABLE 3:** YLL rate of acute hepatitis among children under five by sex and location in Ethiopia, 1990 to 2023.

Location	Sex	Acute hepatitis	Annualized rate of change	Hepatitis A	Annualized rate of change	Hepatitis B	Annualized rate of change	Hepatitis C	Annualized rate of change	Hepatitis E	Annualized rate of change
Addis Ababa	Male	239 (96, 459)	0.14 (−0.63, 1.91)	4 (0, 35)	−0.86 (−0.99, −0.38)	226 (86, 430)	0.32 (−0.58, 2.72)	0 (0, 0)	−0.20 (−0.88, 9.37)	39 (2, 98)	−0.04 (−0.89, 5.92)
	Female	48 (18, 95)	−0.47 (−0.87, 0.89)	8 (0, 32)	−0.81 (−0.98, −0.04)	22 (4, 54)	−0.25 (−0.78, 3.71)	1 (0, 8)	−0.02 (−0.92, 8.71)	32 (4, 71)	0.10 (−0.77, 3.32)
	Both	145 (70, 247)	−0.03 (−0.58, 1.15)	6 (1, 23)	−0.83 (−0.98, −0.24)	126 (55, 225)	0.25 (−0.53, 2.19)	1 (0, 4)	−0.04 (−0.89, 8.33)	13 (3, 37)	0.04 (−0.71, 2.30)

Afar	Male	544 (198, 1120)	−0.05 (−0.73, 1.80)	144 (40, 378)	−0.58 (−0.91, 0.96)	390 (124, 831)	0.69 (−0.57, 13.67)	0 (0, 1)	−0.24 (−0.95, 13.04)	83 (13, 213)	0.42 (−0.71, 8.04)
	Female	622 (220, 1285)	−0.16 (−0.77, 1.73)	340 (80, 845)	−0.32 (−0.86, 2.41)	199 (28, 484)	0.29 (−0.79, 6.66)	17 (1, 61)	−0.26 (−0.87, 11.22)	47 (12, 107)	−0.01 (−0.75, 6.97)
	Both	581 (274, 976)	−0.11 (−0.62, 1.23)	237 (78, 477)	−0.43 (−0.83, 0.58)	300 (111, 546)	0.54 (−0.50, 5.50)	8 (1, 29)	−0.24 (−0.87, 11.00)	15 (3, 45)	0.06 (−0.69, 4.43)

Amhara	Male	551 (222, 1138)	−0.21 (−0.74, 1.42)	259 (84, 615)	−0.53 (−0.88, 0.51)	267 (83, 582)	1.07 (−0.62, 18.73)	0 (0, 0)	0.34 (−0.95, 48.46)	49 (9, 118)	1.22 (−0.51, 12.86)
	Female	274 (96, 516)	−0.31 (−0.80, 1.49)	167 (55, 371)	−0.49 (−0.87, 0.96)	61 (2, 177)	0.42 (−0.86, 15.34)	7 (0, 28)	0.26 (−0.90, 32.50)	32 (10, 66)	0.84 (−0.73, 25.29)
	Both	415 (219, 766)	−0.24 (−0.69, 0.89)	214 (107, 389)	−0.52 (−0.82, 0.26)	166 (50, 335)	0.92 (−0.58, 8.44)	3 (0, 14)	0.26 (−0.90, 32.04)	10 (2, 25)	0.97 (−0.46, 10.47)

Benishangul‐Gumuz	Male	640 (268, 1362)	0.15 (−0.67, 2.89)	139 (32, 400)	−0.57 (−0.91, 1.28)	489 (186, 970)	1.12 (−0.54, 19.79)	0 (0, 1)	0.46 (−0.89, 21.41)	38 (9, 84)	0.99 (−0.55, 11.71)
	Female	506 (170, 1024)	−0.24 (−0.81, 2.17)	211 (52, 523)	−0.52 (−0.90, 3.14)	183 (28, 486)	0.43 (−0.83, 10.50)	29 (2, 95)	−0.08 (−0.87, 18.09)	23 (8, 46)	0.28 (−0.72, 9.80)
	Both	574 (277, 931)	−0.06 (−0.63, 1.69)	174 (71, 355)	−0.54 (−0.85, 0.70)	340 (138, 625)	0.89 (−0.48, 8.14)	14 (1, 46)	−0.09 (−0.87, 17.55)	13 (3, 42)	0.33 (−0.64, 6.68)

Dire Dawa	Male	442 (154, 957)	0.07 (−0.72, 2.14)	53 (11, 184)	−0.73 (−0.95, 0.27)	374 (125, 779)	0.82 (−0.59, 6.85)	0 (0, 0)	0.19 (−0.88, 12.99)	33 (6, 77)	0.66 (−0.65, 9.78)
	Female	206 (60, 439)	−0.28 (−0.83, 2.22)	64 (10, 189)	−0.65 (−0.95, 1.24)	86 (7, 230)	0.80 (−0.71, 10.53)	7 (0, 33)	−0.35 (−0.95, 13.88)	23 (8, 42)	0.04 (−0.74, 5.52)
	Both	327 (140, 623)	−0.07 (−0.68, 1.22)	59 (18, 140)	−0.69 (−0.92, 0.21)	234 (85, 486)	0.83 (−0.52, 5.24)	3 (0, 16)	−0.35 (−0.94, 12.99)	11 (2, 33)	0.13 (−0.63, 3.51)

Ethiopia	Male	286 (117, 564)	−0.42 (−0.81, 0.72)	90 (30, 225)	−0.71 (−0.92, 0.01)	186 (74, 363)	0.07 (−0.73, 5.30)	0 (0, 0)	−0.40 (−0.94, 7.10)	42 (7, 98)	0.38 (−0.68, 6.79)
	Female	190 (66, 377)	−0.62 (−0.89, 0.36)	99 (31, 230)	−0.71 (−0.93, 0.28)	48 (8, 126)	−0.37 (−0.88, 2.99)	5 (1, 19)	−0.71 (−0.96, 4.67)	26 (8, 55)	−0.36 (−0.83, 3.08)
	Both	239 (129, 425)	−0.52 (−0.79, 0.08)	95 (44, 172)	−0.71 (−0.90, −0.20)	119 (49, 219)	−0.06 (−0.70, 2.10)	3 (0, 9)	−0.71 (−0.96, 4.60)	3 (0, 14)	−0.28 (−0.77, 1.95)

Gambella	Male	393 (152, 795)	−0.04 (−0.78, 2.04)	50 (9, 169)	−0.79 (−0.97, 0.31)	329 (121, 689)	1.03 (−0.60, 15.15)	0 (0, 1)	0.49 (−0.86, 20.86)	26 (8, 56)	1.27 (−0.52, 16.51)
	Female	187 (57, 426)	−0.48 (−0.88, 1.48)	72 (14, 181)	−0.70 (−0.96, 1.58)	75 (5, 209)	0.58 (−0.87, 10.02)	7 (0, 28)	−0.69 (−0.98, 7.03)	14 (6, 28)	−0.36 (−0.89, 5.31)
	Both	293 (140, 517)	−0.24 (−0.74, 0.91)	61 (20, 133)	−0.75 (−0.94, −0.08)	206 (87, 394)	0.95 (−0.52, 7.81)	3 (0, 14)	−0.69 (−0.97, 6.85)	11 (3, 32)	−0.19 (−0.78, 3.32)

Harari	Male	369 (133, 758)	0.32 (−0.64, 3.41)	24 (3, 113)	−0.80 (−0.97, 0.13)	334 (112, 708)	1.20 (−0.43, 8.45)	0 (0, 1)	0.50 (−0.91, 13.66)	15 (3, 35)	0.59 (−0.67, 9.26)
	Female	181 (52, 424)	−0.17 (−0.80, 2.14)	62 (10, 184)	−0.56 (−0.93, 1.16)	66 (6, 192)	0.98 (−0.66, 9.87)	11 (1, 48)	0.13 (−0.89, 14.88)	13 (5, 23)	0.19 (−0.73, 6.74)
	Both	278 (128, 484)	0.11 (−0.59, 1.72)	42 (11, 97)	−0.67 (−0.93, 0.00)	204 (86, 399)	1.16 (−0.37, 6.13)	5 (0, 23)	0.13 (−0.88, 14.51)	5 (1, 14)	0.26 (−0.65, 3.73)

Oromia	Male	170 (67, 364)	−0.49 (−0.84, 0.62)	24 (6, 64)	−0.85 (−0.96, −0.24)	143 (54, 293)	−0.19 (−0.77, 3.12)	0 (0, 0)	−0.71 (−0.95, 1.93)	30 (6, 66)	−0.25 (−0.83, 4.54)
	Female	112 (38, 221)	−0.69 (−0.91, 0.15)	51 (16, 108)	−0.79 (−0.96, 0.02)	32 (8, 76)	−0.48 (−0.90, 3.50)	3 (1, 9)	−0.70 (−0.96, 6.82)	17 (6, 35)	−0.47 (−0.85, 1.70)
	Both	142 (71, 234)	−0.59 (−0.83, −0.09)	37 (14, 72)	−0.81 (−0.94, −0.42)	89 (40, 165)	−0.26 (−0.77, 1.52)	1 (0, 4)	−0.71 (−0.96, 6.71)	21 (5, 61)	−0.45 (−0.83, 1.49)

Sidama	Male	177 (70, 353)	−0.23 (−0.77, 1.35)	74 (22, 176)	−0.54 (−0.88, 0.90)	92 (27, 195)	0.40 (−0.68, 15.38)	0 (0, 0)	0.02 (−0.84, 14.31)	39 (8, 93)	1.40 (−0.41, 14.07)
	Female	77 (23, 172)	−0.46 (−0.88, 1.40)	51 (15, 125)	−0.53 (−0.90, 1.69)	11 (1, 32)	−0.11 (−0.91, 8.34)	1 (0, 4)	−0.75 (−0.98, 3.85)	30 (10, 61)	−0.25 (−0.82, 5.76)
	Both	128 (62, 228)	−0.32 (−0.75, 0.70)	63 (27, 123)	−0.53 (−0.83, 0.34)	52 (18, 104)	0.33 (−0.68, 7.08)	0 (0, 2)	−0.75 (−0.98, 3.75)	13 (3, 38)	0.08 (−0.66, 3.89)

Somali	Male	167 (62, 345)	−0.41 (−0.84, 0.82)	72 (20, 174)	−0.58 (−0.89, 0.94)	90 (25, 203)	−0.18 (−0.82, 5.23)	0 (0, 0)	−0.61 (−0.97, 7.27)	69 (13, 172)	0.27 (−0.73, 8.00)
	Female	176 (59, 376)	−0.56 (−0.88, 0.52)	106 (28, 291)	−0.60 (−0.91, 1.33)	34 (6, 86)	−0.50 (−0.92, 2.22)	5 (0, 18)	−0.74 (−0.96, 2.45)	40 (12, 87)	−0.38 (−0.87, 3.74)
	Both	171 (83, 282)	−0.50 (−0.79, 0.18)	88 (37, 177)	−0.59 (−0.84, 0.15)	63 (22, 126)	−0.30 (−0.81, 2.27)	3 (0, 9)	−0.74 (−0.96, 2.49)	7 (1, 22)	−0.32 (−0.79, 2.63)

Southwest	Male	479 (179, 986)	−0.39 (−0.82, 0.83)	146 (37, 406)	−0.65 (−0.92, 0.82)	311 (106, 650)	−0.14 (−0.80, 5.51)	0 (0, 0)	−0.44 (−0.92, 7.14)	51 (11, 112)	1.02 (−0.54, 11.51)
	Female	180 (55, 391)	−0.62 (−0.91, 0.75)	101 (26, 248)	−0.68 (−0.94, 1.34)	35 (4, 96)	−0.57 (−0.94, 2.84)	5 (0, 21)	−0.79 (−0.98, 3.55)	29 (9, 59)	−0.28 (−0.85, 6.64)
	Both	332 (155, 606)	−0.48 (−0.80, 0.31)	124 (51, 262)	−0.66 (−0.89, 0.17)	175 (65, 341)	−0.21 (−0.81, 2.31)	2 (0, 10)	−0.78 (−0.98, 3.42)	39 (2, 98)	−0.07 (−0.72, 3.28)

SNNP	Male	277 (105, 596)	−0.63 (−0.88, 0.17)	100 (32, 280)	−0.78 (−0.94, −0.12)	164 (54, 347)	−0.44 (−0.87, 2.71)	0 (0, 0)	−0.71 (−0.97, 2.96)	32 (4, 71)	0.25 (−0.68, 5.61)
	Female	264 (92, 540)	−0.78 (−0.93, −0.23)	138 (40, 320)	−0.82 (−0.96, 0.00)	51 (4, 143)	−0.72 (−0.97, 0.72)	6 (0, 25)	−0.90 (−0.99, 0.81)	13 (3, 37)	−0.60 (−0.90, 2.04)
	Both	271 (145, 478)	−0.72 (−0.88, −0.33)	119 (51, 233)	−0.80 (−0.94, −0.31)	109 (42, 209)	−0.54 (−0.87, 0.74)	3 (0, 12)	−0.90 (−0.99, 0.79)	83 (13, 213)	−0.55 (−0.85, 1.30)

Tigray	Male	324 (132, 669)	0.66 (−0.47, 3.90)	36 (8, 110)	−0.69 (−0.94, 0.62)	280 (112, 541)	2.58 (−0.16, 18.64)	0 (0, 0)	1.26 (−0.78, 34.25)	47 (12, 107)	1.45 (−0.46, 12.95)
	Female	259 (94, 499)	−0.43 (−0.83, 1.23)	96 (21, 245)	−0.69 (−0.94, 0.84)	100 (15, 242)	0.35 (−0.78, 9.28)	12 (1, 47)	−0.29 (−0.91, 13.66)	15 (3, 45)	0.04 (−0.75, 8.06)
	Both	292 (151, 506)	−0.09 (−0.65, 1.36)	65 (21, 133)	−0.69 (−0.92, 0.22)	192 (89, 376)	1.52 (−0.28, 8.25)	6 (0, 23)	−0.29 (−0.91, 13.52)	49 (9, 118)	0.13 (−0.68, 4.89)

Hepatitis A accounted for an estimated 95 YLLs per 100,000 children under five nationally in 2023, with similar rates between sexes and lower rates in Addis Ababa (Table 3). A statistically significant decline was observed nationally from 1990 to 2023, with an ARC of −0.71% (95% UI: −0.90, −0.20) per year, with the largest reductions in Addis Ababa, Gambella, Oromia, and SNNP (Table 3).

Hepatitis B was estimated to account for 119 YLLs per 100,000 children under five (95% UI: 52, 217) nationally in 2023, with the rate comparable across regions except for lower rates among females in Addis Ababa. No significant trend changes were observed from 1990 to 2023, with an ARC of −0.06% (95% UI: −0.70, 2.10) per year (Table 3).

Hepatitis C virus infection caused an estimated 8 YLLs per 100,000 children under five (95% UI: 1, 29) in Afar and 14 YLLs per 100,000 children under five (95% UI: 1, 46) in Benishangul‐Gumuz in 2023. From 1990 to 2023, there were no statistically significant differences in the rates of YLLs at all levels (Table 3).

There were an estimated 3 YLLs per 100,000 children under five (95% UI: 0, 14) from Hepatitis E virus infections in 2023, with 10 YLLs per 100,000 males (95% UI: 2, 25) and 39 YLLs per 100,000 females (95% UI: 9, 89). While the point estimates suggest a higher burden among females, the wide and overlapping 95% UIs indicate that this observed difference is not statistically robust (Table 3).

### 3.4. Premature Mortality From Acute Viral Hepatitis Among Infants

**FIGURE 5:**
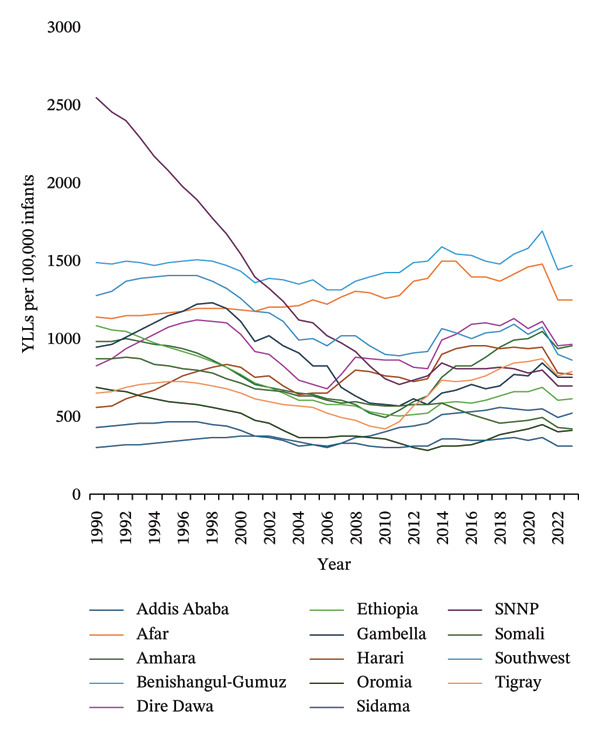
YLL rates of acute hepatitis among infants in Ethiopia, 1990 to 2023.

There were an estimated 604 YLLs from AVH (95% UI: 321, 1062) among 100,000 infants in Ethiopia in 2023. Hepatitis A accounted for 164 YLLs (95% UI: 77, 313), Hepatitis B for 331 YLLs (95% UI: 152, 641), Hepatitis C for 12 YLLs (95% UI: 2, 44), and Hepatitis E for 97 YLLs (95% UI: 34, 198) per 100,000 infants. Rates were similar across subnational regions, with the exception of lower hepatitis A YLLs in Addis Ababa (11 per 100,000 [95% UI: 1, 56]). From 1990 to 2023, no statistically significant trend changes were observed nationally or subnationally for any type of hepatitis. Detailed estimates by region are presented in Table 4 (Figure 5).

**TABLE 4:** YLL rate of acute viral hepatitis among infants by location in Ethiopia, 1990 to 2023.

Location	Acute hepatitis	Annualized rate of change	Hepatitis A	Annualized rate of change	Hepatitis B	Annualized rate of change	Hepatitis C	Annualized rate of change	Hepatitis E	Annualized rate of change
	2023	1990–2023	2023	1990–2023	2023	1990–2023	2023	1990–2023	2023	1990–2023
Addis Ababa	517 (254, 886)	0.82 (−0.30, 3.23)	11 (1, 56)	−0.74 (−0.97, 0.36)	450 (184, 832)	1.28 (−0.20, 4.88)	4 (0, 20)	0.14 (−0.89, 10.27)	52 (15, 106)	0.31 (−0.67, 3.12)
Afar	1241 (577, 2043)	0.16 (−0.49, 2.01)	319 (110, 746)	−0.46 (−0.84, 0.53)	732 (277, 1441)	1.46 (−0.21, 11.46)	38 (2, 134)	−0.19 (−0.86, 12.25)	151 (39, 311)	0.10 (−0.70, 5.58)
Amhara	944 (503, 1613)	0.00 (−0.61, 1.79)	385 (184, 733)	−0.47 (−0.82, 0.49)	425 (157, 866)	1.76 (−0.40, 16.12)	16 (0, 64)	0.43 (−0.89, 37.48)	118 (15, 267)	1.16 (−0.49, 14.31)
Benishangul‐Gumuz	1466 (700, 2512)	0.04 (−0.61, 1.98)	261 (94, 639)	−0.64 (−0.89, 0.56)	938 (418, 1814)	0.94 (−0.49, 9.68)	65 (4, 217)	0.05 (−0.85, 20.63)	202 (48, 475)	0.48 (−0.61, 8.27)
Dire Dawa	953 (425, 1854)	0.23 (−0.62, 2.03)	92 (27, 224)	−0.72 (−0.93, 0.21)	712 (275, 1470)	1.22 (−0.42, 7.67)	16 (1, 76)	−0.25 (−0.94, 15.65)	132 (40, 274)	0.26 (−0.62, 4.36)
Ethiopia	604 (321, 1062)	−0.42 (−0.74, 0.37)	164 (77, 313)	−0.72 (−0.91, −0.11)	331 (152, 641)	0.16 (−0.64, 3.35)	12 (1, 44)	−0.68 (−0.96, 5.26)	97 (34, 198)	−0.24 (−0.76, 2.64)
Gambella	747 (352, 1379)	−0.16 (−0.71, 1.09)	90 (26, 232)	−0.80 (−0.95, −0.07)	549 (230, 1106)	0.94 (−0.56, 8.56)	16 (1, 63)	−0.64 (−0.97, 8.23)	92 (29, 175)	−0.15 (−0.79, 5.15)
Harari	763 (352, 1347)	0.45 (−0.45, 2.44)	60 (13, 165)	−0.71 (−0.95, 0.00)	568 (223, 1157)	1.58 (−0.27, 8.08)	25 (1, 113)	0.30 (−0.87, 17.09)	109 (30, 233)	0.40 (−0.64, 5.08)
Oromia	401 (208, 660)	−0.40 (−0.77, 0.43)	60 (17, 133)	−0.79 (−0.94, −0.20)	271 (121, 518)	0.07 (−0.69, 3.45)	6 (1, 20)	−0.68 (−0.95, 7.67)	63 (24, 125)	−0.40 (−0.82, 1.78)
Sidama	303 (147, 550)	−0.25 (−0.74, 0.90)	109 (42, 244)	−0.58 (−0.87, 0.37)	144 (47, 300)	0.49 (−0.67, 9.17)	2 (0, 9)	0.72 (−0.98, 4.38)	48 (18, 91)	0.06 (−0.67, 5.10)
Somali	417 (207, 673)	−0.50 (−0.77, 0.16)	162 (69, 349)	−0.65 (−0.88, 0.00)	170 (61, 338)	−0.22 (−0.77, 2.51)	12 (1, 40)	−0.73 (−0.96, 2.67)	73 (23, 153)	−0.31 (−0.79, 3.01)
Southwest	859 (386, 1556)	−0.29 (−0.74, 0.85)	234 (86, 520)	−0.63 (−0.88, 0.29)	497 (190, 1022)	0.21 (−0.73, 5.29)	11 (1, 47)	−0.74 (−0.98, 4.43)	117 (38, 244)	0.02 (−0.72, 4.85)
SNNP	692 (374, 1191)	−0.72 (−0.87, −0.31)	215 (89, 477)	−0.83 (−0.95, −0.36)	291 (110, 576)	−0.55 (−0.88, 0.80)	14 (1, 57)	−0.89 (−0.99, 1.02)	172 (46, 388)	−0.52 (−0.84, 1.77)
Tigray	781 (412, 1394)	0.27 (−0.50, 2.09)	90 (30, 222)	−0.72 (−0.94, 0.07)	537 (249, 1031)	2.50 (−0.06, 12.35)	29 (2, 108)	−0.20 (−0.90, 15.45)	126 (35, 264)	0.21 (−0.69, 6.14)

## 4. Discussion

This study aimed to evaluate the trends in incidence and premature mortality from AVH among children under five in Ethiopia from 1990 to 2023. The results demonstrate a significant decline in Hepatitis B incidence, contrasting with slower progress for Hepatitis A and E. Premature mortality also decreased nationally, though regional disparities persisted. These findings underscore the need for tailored interventions to address subnational variations and the differing epidemiology of hepatitis subtypes.

The decline in Hepatitis B incidence is plausibly associated with the implementation of Hepatitis B vaccination, especially among newborns and infants. Ethiopia introduced the Hepatitis B vaccine as part of the pentavalent vaccine (combined diphtheria–tetanus–pertussis–Hepatitis B–Haemophilus influenzae Type b) in the Expanded Program on Immunization (EPI) in 2007. The WHO–UNICEF estimates of pentavalent vaccine coverage in Ethiopia increased from approximately 30% in 2007 to over 70% by 2019 [18, 19]. The temporal coincidence of this coverage expansion with the observed decline in Hepatitis B incidence among children under five supports this interpretation, though our ecological study design does not permit definitive causal attribution.

The greater reductions seen in urban areas such as Addis Ababa may reflect enhanced access to immunization services, better sanitation, and improved public health infrastructure [16, 20], whereas rural regions may lag due to logistical challenges in vaccine distribution. To sustain progress, vaccination programs should be strengthened in underserved areas, and catch‐up campaigns for older children should be considered [21]. Despite being the most incident, Hepatitis A and E showed minimal declines, suggesting persistent environmental and infrastructural challenges. These viruses primarily spread through contaminated water and poor sanitation [22], which remain widespread in many regions. Public health efforts should prioritize improving water quality, hygiene education, and sanitation infrastructure [7, 23], particularly in high‐burden areas like Afar and Somali, where incidence rates remain elevated.

While premature mortality declined nationally, regions such as Afar and Benishangul‐Gumuz exhibited higher point estimates for YLL rates, likely due to limited healthcare access and delayed case management. However, the overlapping UIs suggest these differences may not be statistically robust, emphasizing the need for caution in interpreting regional rankings. The wide UIs in modeled estimates for low‐burden outcomes preclude definitive conclusions about regional disparities [24].

Male children exhibited higher point estimates for YLL rates than females in some analyses, though the UIs overlapped substantially. These wide UIs, characteristic of modeled estimates in low‐burden settings, prevent firm conclusions about sex differences. While the point estimates suggest possible disparities, the statistical imprecision means we cannot definitively conclude that male and female children experience different premature mortality burdens from AVH. Biological factors, healthcare‐seeking behavior, or differential exposure risks may contribute to the observed point estimates, but further research with individual‐level data is needed to explore these potential disparities and equitable healthcare delivery [21, 23].

The overlapping clinical presentations of viral hepatitis with other endemic infectious diseases in Ethiopia, such as visceral leishmaniasis, can complicate diagnosis and management, particularly in resource‐limited settings where diagnostic capacity is constrained [25]. This underscores the importance of strengthening diagnostic infrastructure alongside prevention and treatment efforts.

An important observation from our analysis is the pattern of YLL rates in several regions, which show an increase between approximately 2010 and 2021 before declining toward the end of the study period. This pattern may reflect several factors: the complex interplay of conflict and displacement affecting healthcare access in some regions, the potential impact of the COVID‐19 pandemic on health service utilization, or the time lag between vaccination introduction and measurable mortality reduction [26]. For example, the Tigray region experienced conflict starting in late 2020, which may have disrupted healthcare services and contributed to the observed trends. In the Somali and Afar regions, recurrent drought and population mobility may have affected both disease transmission and healthcare access. These findings highlight the vulnerability of health gains to social and environmental disruptions and underscore the need for resilient health systems that can maintain essential services during crises. However, given the wide UIs, these regional patterns should be interpreted with caution and call for further investigation.

Overall, while Ethiopia has made progress in reducing the burden of AVH among children under five, the findings of this study reinforce the need for targeted strategies addressing regional disparities and the specific epidemiology of hepatitis subtypes [27, 28].

The findings highlight the success of Hepatitis B vaccination but also reveal gaps in addressing Hepatitis A and E. A multipronged approach, combining vaccination, sanitation improvements, and health system strengthening, is critical. Policymakers should prioritize region‐specific strategies, particularly in high‐burden areas, to accelerate progress toward hepatitis elimination [21, 29].

This study has several limitations. First, the estimates rely on modeled data from the GBD study, which incorporates assumptions and may not capture all subnational variations accurately. Second, underreporting and misclassification of hepatitis types, especially in rural or conflict‐affected areas, could influence results. Third, the study could not assess coinfections or interactions with other childhood illnesses, which might compound the burden of hepatitis. Lastly, while the study trends by age, sex, and region, it lacked individual‐level data to explore socioeconomic or behavioral risk factors that may influence exposure and outcomes, and there are no data on the YLL rate under 28 days.

## 5. Conclusion

In conclusion, this subnational analysis of GBD 2023 data reveals a significant national decline in both the incidence and premature mortality from AVH among children under five in Ethiopia from 1990 to 2023, particularly for Hepatitis B. While the burden has decreased overall, regional and sex‐specific disparities persist, though most were not statistically robust due to overlapping 95% UIs. Sustained efforts are needed to strengthen vaccination programs, improve sanitation and water infrastructure, and enhance equitable healthcare access, especially in regions where the burden remains high. These findings underscore the importance of regionally targeted public health strategies to accelerate hepatitis elimination among vulnerable child populations.

## Author Contributions

Sebsibe Tadesse: conceptualization, methodology, formal analysis, data curation, visualization, writing–original draft, writing–review and editing, supervision.

Medhin Mehari: methodology, formal analysis, data curation, visualization, writing–original draft, writing–review and editing, supervision.

Jemal Bedewi: validation, investigation, writing–review and editing.

Zufan Tessema: validation, investigation, writing–review and editing.

Abadi Hailay Atsbaha: validation, investigation, writing–review and editing.

Alqeer Aliyo: validation, investigation, writing–review and editing.

Miesa Gelchu: validation, investigation, writing–review and editing.

Tibeso Gemechu: validation, investigation, writing–review and editing.

Yohannes Fekadu: validation, investigation, writing–review and editing.

Girma Ashenafi: validation, investigation, writing–review and editing.

## Funding

This study did not receive any specific funding.

## Disclosure

All authors have read and approved the final version of the manuscript. All authors were involved in the analysis and interpretation of the data. Medhin Mehari had full access to all of the data in the study and takes complete responsibility for the integrity of the data and the accuracy of the data analysis.

The authors have verified that none of the cited references in this manuscript have been retracted. For references with published corrections, we have evaluated these and confirmed that they do not affect the relevance or interpretation of the citations in this work.

## Conflicts of Interest

The authors declare no conflicts of interest.

## Supporting Information

Additional supporting information can be found online in the Supporting Information section.

## Supporting information


**Supporting Information** STROBE_Checklist_Medhin_Hep_Ethiopia.

## Data Availability

The data that support the findings of this study are publicly available from the Global Burden of Diseases, Injuries, and Risk Factors Study (GBD) Results Tool (https://vizhub.healthdata.org/gbd-results/). The specific GBD 2023 estimates used in this analysis can be accessed by selecting the following parameters: cause (acute viral hepatitis), location (Ethiopia and subnational regions), age groups (under 5, infant, neonatal), sex (male, female, both), metrics (incidence and YLLs), and year range (1990–2023). All data analyzed in this study are derived from these publicly available sources.
