# Supplementary material for: Trends in Incidence and Premature Mortality (Years of Life Lost) From Acute Viral Hepatitis Among Children Under Five in Ethiopia, From 1990 to 2023: A Global Burden of Disease 2023 Study
Source: Glob Health Epidemiol Genom. 2026 Aug 12;2026:5287334. doi: 10.1155/ghe3/5287334 (PMC13463311; doi:10.1155/ghe3/5287334)
Supplement: Supplementary file 1 [file GHE3-2026-5287334-s001.doc]

**Completed STROBE Checklist - Cross-Sectional Study**

**Manuscript Title:** Trends in incidence and premature mortality (years of life lost) from acute viral hepatitis among children under five in Ethiopia, from 1990 to 2023: a Global Burden of Disease 2023 study

**Authors:** Medhin Mehari, Jemal Bedewi, Zufan Tessema, Abadi Hailay Atsbaha, Alqeer Aliyo, Miesa Gelchu, Tibeso Gemechu, Yohannes Fekadu, Girma Ashenafi, Sebsibe Tadesse

**Journal:** Global Health, Epidemiology and Genomics

|  | Item No | Recommendation | | | Reported |
| --- | --- | --- | --- | --- | --- |
| **Title and abstract** | 1 | (*a*) Indicate the study’s design with a commonly used term in the title or the abstract | | | Yes |
|  |  | (*b*) Provide in the abstract an informative and balanced summary of what was done and what was found | | | Yes |
| Introduction | | | | |  |
| Background/rationale | 2 | Explain the scientific background and rationale for the investigation being reported | | Yes | |
| Objectives | 3 | State specific objectives, including any prespecified hypotheses | | Yes | |
| Methods | | | |  | |
| Study design | 4 | Present key elements of study design early in the paper | | Yes | |
| Setting | 5 | Describe the setting, locations, and relevant dates, including periods of recruitment, exposure, follow-up, and data collection | | Yes | |
| Participants | 6 | (*a*) Give the eligibility criteria, and the sources and methods of selection of participants | | Yes | |
| Variables | 7 | Clearly define all outcomes, exposures, predictors, potential confounders, and effect modifiers. Give diagnostic criteria, if applicable | | Yes | |
| Data sources/ measurement | 8* | For each variable of interest, give sources of data and details of methods of assessment (measurement). Describe comparability of assessment methods if there is more than one group | Yes | | |
| Bias | 9 | Describe any efforts to address potential sources of bias | Yes | | |
| Study size | 10 | Explain how the study size was arrived at | Yes | | |
| Quantitative variables | 11 | Explain how quantitative variables were handled in the analyses. If applicable, describe which groupings were chosen and why | Yes | | |
| Statistical methods | 12 | (*a*) Describe all statistical methods, including those used to control for confounding | Yes | | |
|  |  | (*b*) Describe any methods used to examine subgroups and interactions | Yes | | |
|  |  | (*c*) Explain how missing data were addressed | Yes | | |
|  |  | (*d*) If applicable, describe analytical methods taking account of sampling strategy | Yes | | |
|  |  | (*e*) Describe any sensitivity analyses | Yes | | |
| Results | | |  | | |
| Participants | 13* | (a) Report numbers of individuals at each stage of study—eg numbers potentially eligible, examined for eligibility, confirmed eligible, included in the study, completing follow-up, and analysed | Yes | | |
|  |  | (b) Give reasons for non-participation at each stage | Yes | | |
|  |  | (c) Consider use of a flow diagram | Yes | | |
| Descriptive data | 14* | (a) Give characteristics of study participants (eg demographic, clinical, social) and information on exposures and potential confounders | Yes | | |
|  |  | (b) Indicate number of participants with missing data for each variable of interest | Yes | | |
| Outcome data | 15* | Report numbers of outcome events or summary measures | Yes | | |
| Main results | 16 | (*a*) Give unadjusted estimates and, if applicable, confounder-adjusted estimates and  their precision (eg, 95% confidence interval). Make clear which confounders were adjusted for and why they were included | Yes | | |
|  |  | (*b*) Report category boundaries when continuous variables were categorized | Yes | | |
|  |  | (*c*) If relevant, consider translating estimates of relative risk into absolute risk for a meaningful time period | Yes | | |
| Other analyses | 17 | Report other analyses done—eg analyses of subgroups and interactions, and sensitivity analyses | Yes | | |
| Discussion | | |  | | |
| Key results | 18 | Summarise key results with reference to study objectives | Yes | | |
| Limitations | 19 | Discuss limitations of the study, taking into account sources of potential bias or imprecision. Discuss both direction and magnitude of any potential bias | Yes | | |
| Interpretation | 20 | Give a cautious overall interpretation of results considering objectives, limitations, multiplicity of analyses, results from similar studies, and other relevant evidence | Yes | | |
| Generalisability | 21 | Discuss the generalisability (external validity) of the study results | Yes | | |
| Other information | | |  | | |
| Funding | 22 | Give the source of funding and the role of the funders for the present study and, if applicable, for the original study on which the present article is based | Yes | | |
